# Correction: Getting funded in a highly fluctuating environment: Shifting from excellence to luck and timing

**DOI:** 10.1371/journal.pone.0301967

**Published:** 2024-04-18

**Authors:** Eneli Kindsiko, Kärt Rõigas, Ülo Niinemets

S1 Dataset is incorrect. Please view the correct S1 Dataset below.

## Supporting information

S1 Dataset (XLSX)
