# Supplementary figures and images for: Getting funded in a highly fluctuating environment: Shifting from excellence to luck and timing
Source: PLoS One. 2022 Nov 7;17(11):e0277337. doi: 10.1371/journal.pone.0277337 (PMC9639839; doi:10.1371/journal.pone.0277337)

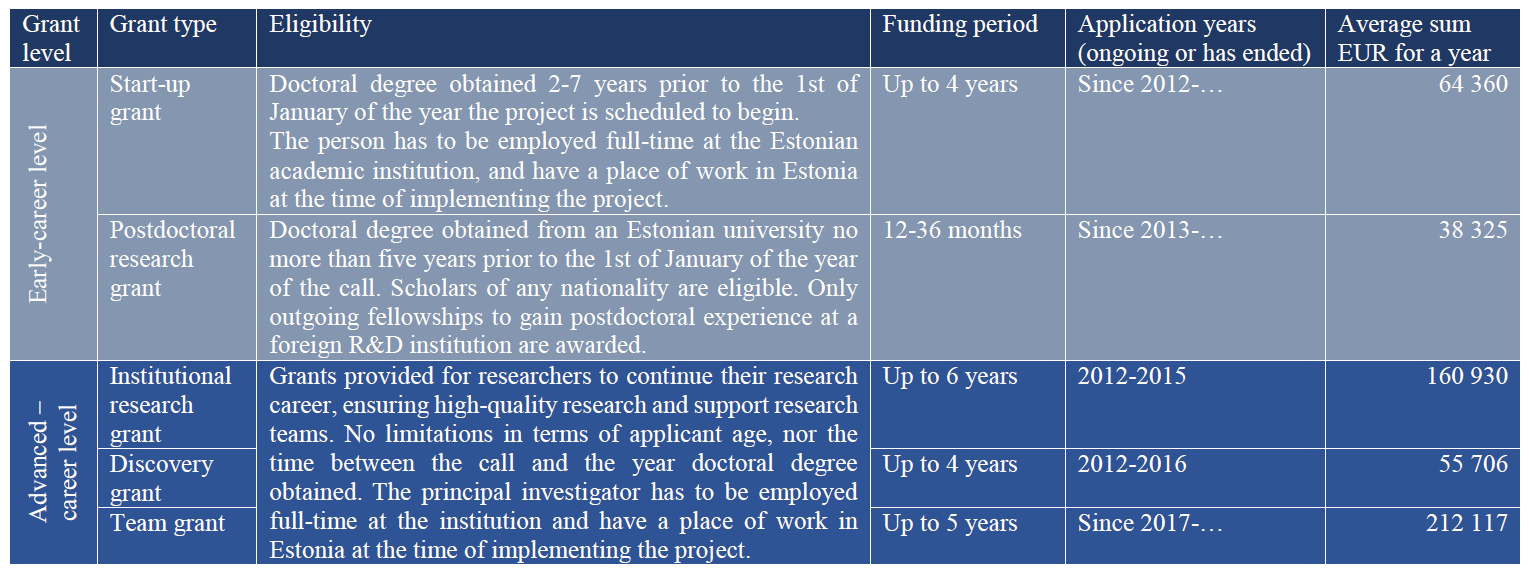

Supplement: S1 Table — (TIF) [file pone.0277337.s001.tif]

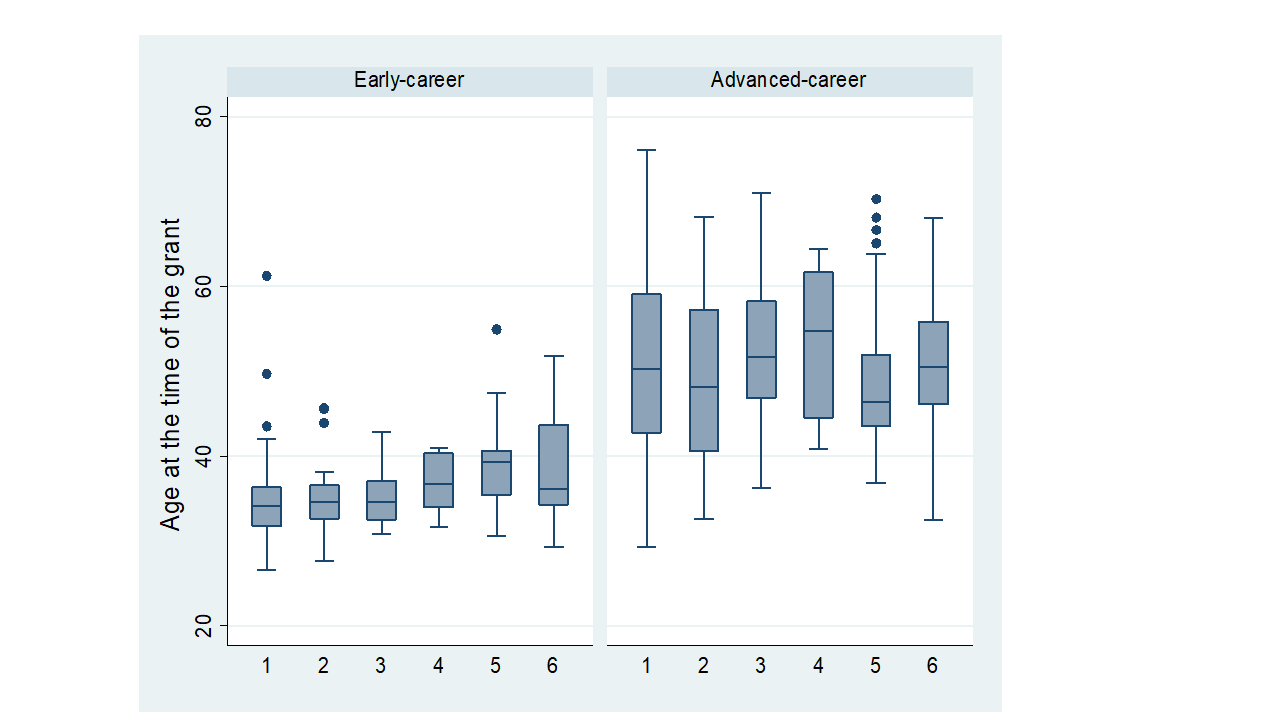

Supplement: S1 Fig — 1 –Natural sciences; 2 –Engineering and technology; 3 –Medical and health sciences; 4 –Agricultural and veterinary sciences; 5 –Social sciences; 6 –Humanities and the arts. (TIF) [file pone.0277337.s003.tif]

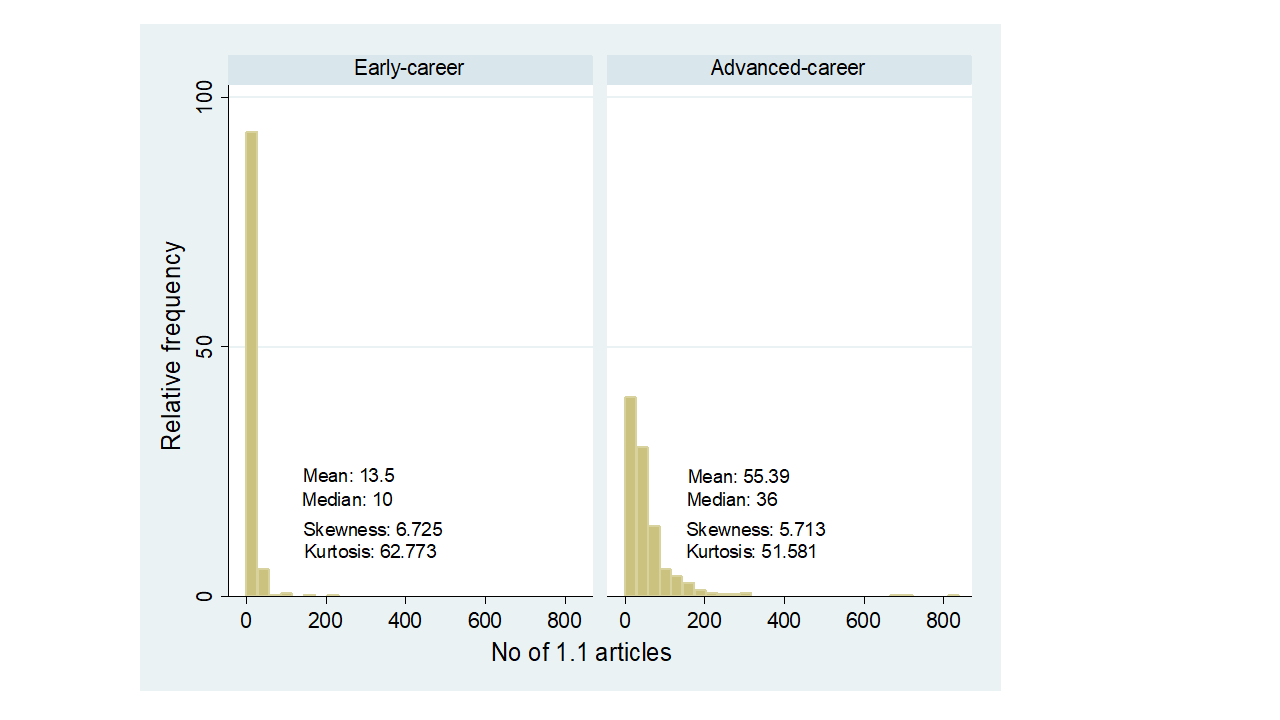

Supplement: S2 Fig — Smaller graphs show the distribution after logarithmic transformation. (TIF) [file pone.0277337.s004.tif]

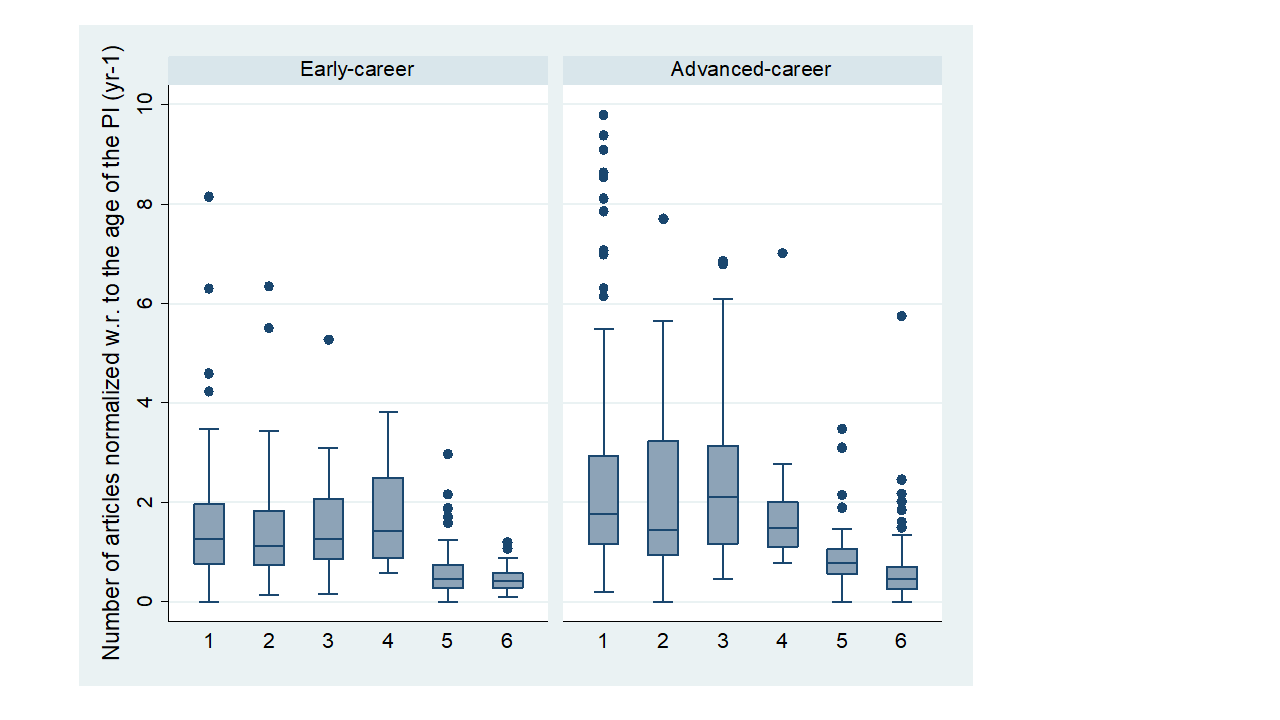

Supplement: S3 Fig — The outliers with the normalized number of articles above 10 are removed to construct the graph. 1 –Natural sciences; 2 –Engineering and technology; 3 –Medical and health sciences; 4 –Agricultural and veterinary sciences; 5 –Social sciences; 6 –Humanities and the arts. (TIF) [file pone.0277337.s005.tif]
