# Supplementary material for: Getting funded in a highly fluctuating environment: Shifting from excellence to luck and timing
Source: PLoS One. 2022 Nov 7;17(11):e0277337. doi: 10.1371/journal.pone.0277337 (PMC9639839; doi:10.1371/journal.pone.0277337)
Supplement: Attachment — Submitted filename: Response to reviewers.docx [file pone.0277337.s006.docx]

**Response to Reviewers**

**Reviewer 1**

**1.1. This article has one strong advantage: it is based on a comprehensive proposal database (all competitive research proposals submitted for funding in Estonia over a nine year period) and corresponding bibliometric data on those submitting these proposals. The statistical analysis of these data is extremely well done (with one very minor concern about using full publication counts instead of fractional counts because of high multi-authorship in the large national physics lab in Estonia). The data provides excellent information about proposal success rates by field. These data are also presented in the broader context of policy changes in Estonia. This is an extremely solid foundation on which to build upon.**

Reply: We are grateful for kind words and will give our best to deliver maximum outcome from this database. Regarding the comment about the concern about “using full publication counts instead of fractional counts because of high multi-authorship in the large national physics lab in Estonia”, we admit the challenge, yet it is impossible to solve it. The problem has been well addressed in literature as it poses severe difficulties for the scientometric analysis (see Tol, 2011, Smith et al., 2020).

In case of multi-authored papers only authors themselves are able to say about the workload. In case of 10 authored paper, the first author may have done 90% of work, yet if taken by equal fractions, s/he would only get 1/10^th^. We also considered taking into an account the ranking of authors, who is the first, second, etc, yet this is where disciplinary differences come in (see Johann & Mayer, 2018). In lab-based disciplines it is common to have a long list of authors, yet in humanities and in social sciences single authored publications are still most widespread (Engels et al., 2012; Mayer 2016). Some get authorship for reasons not connected to the contribution in the paper, but due to practical needs (being the one who has financed the study/head of the lab, needing the article for soon to expected career rise, forthcoming doctoral defence and one article is missing, etc.). The growth in number of authors has been seen to be especially present in case of high impact papers – papers that are perceived as most valuable in terms of academic career (Sahu & Panda 2014), which in turn may signpost a spread of small or non-deserved authorship in journals.

In sum, being the second author in natural sciences has different meaning than being a second author in humanities. Also, depending on the preferences of the research group, sometimes they just list the authors alphabetically, sometimes (though on a much smaller scale) it is done based on seniority. We have added a few explanatory sections to the revised manuscript explaining the difficulties and rationale behind not using fractional authorship.

**1.2. The biggest weakness in the article is that the major claims of the authors (vis-à-vis hyper-competition and luck) are not supported by the data. Following is a clarification of these two concepts; why this reviewer doesn’t see support for the author’s claims about hyper-competition and luck and corresponding implications for going forward.**

**Hyper-competition:**

**The concept of hyper-competition was introduced into the field of management in 2010 by Richard D’Aveni. This concept has a very specific meaning. Basically, it describes a situation where competitive advantages are less sustainable (the rich do not automatically get richer). Hyper-competition is also associated with changes in the broader competitive environment (more rivalry among firms- less long term relationships with suppliers and customers) and detectable changes in the temporal persistence of profitability (a more direct indicator that competitive advantages are less sustainable). An excellent discussion of the concept and supporting literature on hyper-competition can be found on Wikipedia.**

**The authors use of the word ‘hyper-competition’ is rooted in two articles that are outside the field of management: Folcher [2016] and Edwards [2017]. Both articles use the word ‘hyper-competition’ to raise a concern about how competitive advantage in academia has changed. Folcher noted how the current practice of using quantitative (usually bibliometric) indicators for faculty promotion and tenure are “replacing deeper considerations of the quality and novelty of work, as well as substantive care for the societal implications of research”. Edwards is concerned that these quantitative methods are resulting in perverse behavior that, when added to decreased funding rates, will result in unethical behavior by researchers and the de-legitimization of academic research. Their use of the concept of ‘hyper competitive’ is slightly different from D’Aveni. Edwards & Folcher are more concerned with a shift in what determines competitive advantage (from qualitative to quantitative indicators) and the corresponding effect on research outputs (less novelty, less concern for societal needs, more unethical behavior).**

**These are two different uses for the same word. Quantitative indicators for the promotion of academics might actually result in competitive advantages that are more sustainable over time- not less sustainable. Edwards & Folcher are actually describing conditions where the basis for success is changing- they are not using the original definition of the concept. These are important distinctions to make going forward**.

Reply: We acknowledge the above stated difficulties with the terms luck and hypercompetition. In the paper we built on these terms because a great share of research talking about grant success seems to do the same (e.g. Moore et al, 2017; Edwards & Roy, 2017; Alberts et al., 2014; Doyle et al, 2021). One explanation may be that there are field specific differences in how these terms have been put in use. For example, most of these papers do not give a definition of hypercompetitive grant system, they claim a system being hypercompetitive based on low percentage of grant receivers, coupled with odd paradox – from one side competitive grant systems expect novel ideas, yet I order to gain funding for investigating these ideas, one should already have excellent metrics and previous success in investigating the idea. To cite this paradox, “hypercompetition results in a system that preferentially funds reliable research, rather than challenging research” (Watanabe, 2014).

Papers that use the terms luck and hypercompetition when discussing the research grant systems, have been also published in top journals like Nature (e.g. Moore et al., 2017), PNAS (e.g. Alberts et al. 2014), and PlosONE (e.g. Doyle et al., 2021).

That said, we fully agree with the reviewer in stating how Edwards and Folcher together with the many listed authors that address competitive research funding “are actually describing conditions where the basis for success is changing- they are not using the original definition of the concept”, that is the concept that emerged from the management in 2010. To be honest, in most cases they also do not define the term.

As the detailed scoring of each application is not available to us (as to test the luck and hypercompetition aspect in a way recommended by the reviewer), we decided to drop the luck and hypercompetition focus from our problem statement and the theoretical part. The article will focus on the most insightful finding – the state of early career researchers in grant competition. This is also the aspect that both reviewers have positively highlighted throughout the reviews.

**1.3. Luck vs. Excellence (in getting a proposal funded):**

**The authors are concerned about two interrelated issues: the ability to identify excellent proposals and the corresponding effect of funding decisions on the survival of early stage researchers.**

**The ability to evaluate the excellence of a proposal is where luck (vs. excellence) comes into play. One has to look at the distribution of proposal evaluations (by expert and by field) to make this claim. I’m not aware of any publications on this- but anecdotally (which means I’ve had the opportunity to see unpublished data on this question), the distribution is very non-linear. There’s usually a very small group of proposals where all the evaluators consider the proposal as ‘excellent’. There’s also a very small group where there is consensus that the proposal is not. But the vast majority of proposals are in the middle group- there isn’t a consensus that this is an excellent or poor proposal. Luck doesn’t play a role in the two extreme cases: funding decisions are based on expert consensus. Luck plays an important role for the vast majority of proposals where evaluators don’t agree. And it is in the group of ambiguous proposals that secondary criteria (such as gender equity, diversity of funded institutions, diversity in the benefits of research and career stage) can be applied.**

Reply: We explained much of the aspects already in the previous point (regarding the application scores). Estonian Research Council is not willing to share the individual scores given to the grants that have been funded vs. not funded. That said, we have only public data about grant holders who have been successful. We kindly appreciate the remark about survival of early stage researchers – it is indeed the most striking finding! Revised version of paper sets the focus on the state of early career researchers in grant competition. We do not have career tracking data on actual movements in career when not successful in grant competition as the names of non-successful applicant are not public information.

**1.4. Survival deals with whether a researcher in Estonia symbolically lives (gets a 5 year grant so they can continue to do research for another 5 years) or symbolically dies (they don’t get funding after trying two or three times- they have to shift to a different career path). To put this in concrete terms, the authors mention that there were about 3000 researchers and 3800 one-year grants in Estonia (I’m basing this on the reported 766 grants with an average 5 year time period). So over this nine year period, there’s only enough funding to support each researcher for 15 months. This leads to a system that could range anywhere from systemic economic starvation (everybody is given one grant that can support them for 15 months- and this has to last 9 years) to extreme economic and social stratification (a few hundred fully funded researchers; the rest have nothing unless they work for the fully funded researchers). Survival of those just starting their career is of particular concern. It’s not just about the age of the work force. It’s also about whether economic and social power is becoming more concentrated. It's about Folcher's point that novelty (and new ideas) might be driven out of the system.**

Reply: It is fact one of the main findings of the paper - early career researchers take the biggest toll in any kind of dysfunctions of the grants system. We have rewritten the discussion part so that in addition to answering RQ 1-3, we also discuss the unwanted side-effects of a nonstable and RPRT driven grant system.

**1.5. Given these two issues- the authors’ logic seems to be the following. If all proposals across all fields are treated equally (the best proposal gets funding), then one might claim that funding is based on excellence. There will still be deaths- but the survivors represent ‘the best of the best’. But as one goes deeper and deeper into the non-consensus group of proposals, funding decisions are more based on luck. In concrete terms, you are especially lucky if you happen to have been an engineering early career researcher in 2018- when the Estonian government decided to invest more money in research that supports job-creation in the manufacturing sector. As the authors put it- you were in the right place at the right time.**

**Weaknesses of this study:**

**My concern is that the data provided by the authors do not support their central premise of hyper-competition and luck. Lower proposal success rates are not necessarily related to hyper-competition. Building on D’Aveni, there’s no evidence that faculty shifted their efforts from forming alliances with funders to attacking their peers (this is one indicator of hyper-competition) and there’s no evidence that the current way to maintain competitive advantage (i.e. publish more in the peer review articles) is deteriorating. Building on Folcher, there’s no evidence that grant decisions were only based on the quantitative rankings of the reviewers (the authors provided no analysis of the quantitative ranking of the proposals and the role of quantitative vs. qualitative evaluations in making the final funding decision). Building on Folcher, there’s no evidence that researchers are less novel after proposal success rates dropped. Building on Edwards, there no evidence of increased unethical behavior. In other words- the data used in this study doesn’t support the fundamental claim or concerns that the environment is hyper-competitive.**

Reply: As explained earlier, we distracted from using luck and hypercompetition, and revised version focusses narrowly on the state of early career researchers, whilst using advanced career level grants as a comparative background.

**1.6. Nor does the data used by the authors support the central premise of luck. Given the reasonable assumption that, in any field, there is a very small percentage of excellent proposal, a very small percentage of extreme poor proposals and a vast middle ground of non-consensus proposals, low proposal acceptance rates will always result in a higher percentage of excellent proposals. Nor does low proposal acceptance rates automatically result in reduced diversity if there is a sufficient pool of non-consensus proposals by which to implement diversity policies.**

Reply: The authors fully agree with this comment. Revision has been made, based on arguments delivered in previous replies.

**1.7. Recommendations:**

**Moving forward, the authors need to be more precise about what they mean by ‘hyper-competition’ and ‘luck’ and make a far stronger link between their claims and the analyses they provide.**

Reply: Due to ambivalence in how also scholars define the terms (see our discussions above), we gave up using this focus.

**1.8. I suggest that the authors incorporate proposal evaluation scores in their analysis. This is critical if one is going to make claims about luck. I doubt that the final decisions on proposals, where there was expert consensus, significantly differed from the quantitative evaluation. The emphasis needs to be on those proposals where evaluators didn’t agree. This is where choices are closer to being random- and actual choices may be biased in a variety of ways (gender, institutional reputation, economic vs. societal benefit, etc.).**

Reply: We contacted the funding agency and we were given aggregated funding scores, but they are not linkable to the specific application – application codes have been unlinked from the applications, keeping two in separate excels. Also, the agency does not provide application scores from individual reviewers, but we get to see only summarized final scores (where 2-3 reviews have been put together and taken average). That said, we are unable to provide the data on scores in a way that is recommended by the reviewer.

**1.9. More importantly, the authors have an opportunity to provide fundamental insights into the survival strategies of early career researchers. This would require that they shift to the use of survival analysis (which is well supported by STATA- the statistical package they are using). They probably have all of the data that is needed to do this. One possibility is to look at the different survival strategies that early career researchers are using (such as being an individual researcher that tends to work alone and requires very little funding vs. being employed in a biochemistry lab or a massive physics lab that has extremely expensive equipment that needs to be maintained). They would need to define what is really meant by ‘death’. Is it the abandonment of a career path in research? Is it the failure to publish a research paper over the next few years? We know very little about the survival strategies of those that received insufficient funding. What really happens to them? The authors of this paper have the opportunity to provide unique insights into the factors that increase (or decrease) the likelihood that an early career researcher will symbolically die.**

Reply: As brought out in the previous reply, we do not have enough data on the unsuccessful applications, meaning that we are not able to track their career, therefore, we are not able to use survival analysis to identify the factors that increase/decrease the probability to survive.

We would define the death by not gaining funding through losing an income after the contract ends. In Estonia, those not holding teaching roles, but are funded by research projects only are dependent on whether their next grant proposal is successful. In other words, they have fixed contracts. The unique and odd part of Estonian academic system is a remarkable share of academics who do research only. That said, their salaries are covered by various research grants, most often by external ones (e.g. Horizon), where early career researchers are highly dependent on the success of advanced lever researchers as they are able to recruit early career researchers into their projects. From the other side, there are PIs who hold multiple large grants at the same time – this is what we have labelled as recurrent funding – money follows money. Some are very well financed, others struggle to survive. Much of this peculiar state of affairs is due to the fact of very low state funding.

**1.10. In summary, I cannot recommend that this article be published as currently structured. The database and analysis, while excellent, is disconnected from the central claims and concerns about hyper-competition and luck. There’s no evidence that reduced proposal acceptance rates results in hyper-competition using the definition by D’Aveni (a deterioration of how competitive advantage is established), Folcher (reduction in novelty) or Edwards (increased gaming of the system). There’s no evidence that more proposals are funded based on luck (this would require an analysis of the proposal evaluation scores by each evaluator and in each field). There are logical reasons to assume that lower acceptance rates will result in funded proposals that are more likely to be excellent. Diversity does not automatically decline when proposal acceptance rates are lower- it might persist and actually increase if secondary criteria are applied to the non-consensus proposals.**

Reply: The paper has been remarkably changed by leaving out luck and hypercompetition and focusing on the situation of early career researchers within competitive national grant allocation system. The central claims now build on the state of early-career researchers, which is well aligned to the empirical results of the paper.

We would also comment the following claim: “There’s no evidence that more proposals are funded based on luck (this would require an analysis of the proposal evaluation scores by each evaluator and in each field). There are logical reasons to assume that lower acceptance rates will result in funded proposals that are more likely to be excellent.” Lower acceptance rates are witnessed during the years where annual money allocated for grants is also smaller and it is striking how success rate fluctuates between 0-67% in case of early career researchers.

 To add, there is also no agreed fixed share of early vs advanced grants and on average, across years, less early career grants are allocated (their annual sum per grant is also much lower) than advanced grants. Figure below illustrates the point well:

One of our core key takeaways from the paper is that one may apply for early career grant only during limited years (2-7 years after gaining a doctoral degree, so 5 years to apply as grant can not start 7 years after the PhD datum). If this window overlaps with overall low annual state funding, even the most excellent proposals will probably be unsuccessful in grant competition. Additionally, looking at the high fluctuations both in share of grants and success rates, as the final decision-making who gains the grant is made by Estonian based expert panel and evaluation committee, we see this as a possible risk in terms of subjectivity and spread of conflict of interests. All members of the mentioned evaluation bodies are advanced grant level researchers, and often work in same university or research centre, some cases also in same small unit (ca 20-50 employees). Yet, this is a side-topic and will not be set as a main focus of this paper.

**Reviewer 2**

**2.1. The authors claim that hypercompetitive research environments make funding allocations random and are therefore detrimental to research excellence and propose to examine this claim in Estonia. To support their claim, the authors collated data from public databases listing details of the awards and of the awardees (via http://www.etag.se) and PI demographic information (via http://www.eetis.ee).**

**The issue of whether research grant funding allocations are promoting research excellence is very topical and highly significant, especially in the tumultuous economic times we are currently experiencing. To examine this in a Northern European Country such as Estonia, is also unusual and could add a diversity of data and perspectives, which the field of research policy needs.**

**A first major issue is the fact that the claims were not well contextualised. There is no review of what hyper-competition in grant funding allocations means, how it has been identified in the past literature, and how it manifests itself in the current research policy landscape: is it the same in all regions of the world? The authors seem to imply that low success rates are the evidence for hyper-competition but this is a little too simplistic as a definition (low success rates could result from an increase in the volume of applications, an increase in the quality threshold, an increase in budgetary constraints, and so forth). The second part of the claim is that hyper competition turn funding allocations in a lottery (by making them "random"). Again, it is not clear what past evidence can support this claim. It may "feel" this way to those who are not successful but this does not mean that the funding decisions allocations are random. For example, the authors claim that decisions are made based on very small differences in scores, but do not review research which can warrant this claim.**

Reply: We have left out the hypercompetition as an anchor for our study. Access to various data provided by funding agencies conserving the evaluation scores *etc* are different based on countries. For example, some studies (e.g. from Netherlands, see van den Besselaar and Sandström, 2015) have been able to analyse even individual reviews, whereas we were denied by Estonian Research Council from connecting even summarized scores with funded projects. That said, the data access differs vastly by countries. For this reason we have handicraft an unique database (based on ETIS) that gather various information about the successful grant receivers from public databases as to cover both socio-demographic aspects, but also to reveal their previous research success (previous funding + number of published articles).

To add, some countries give even access to the full names of non-funded applicants. A good case is again the study by van den Besselaar and Sandström (2015) that revealed career data of early career researchers after being (non)successful in gaining a grant – namely those who got the grant, rose in academic career ladder, those who did not the grant, did not enjoy career rise. Unfortunately we are not able to gain the data about non-successful applicants, thus we are not able to craft the survival analysis of the early-career researchers.

In sum, we restructured the article to take the best out of existing data, whilst not sacrificing novel insights to existing stock of scholarly knowledge.

**2.2. The last part of the claim is that hyper competition and random allocations of funding are detrimental to research excellence. Here again, there is no clear contextualisation of that research excellence means, and whether there is past evidence to show that the standard approach to fund research (e.g., via individual peer-reviewers informing panels and the grant funders) is actually detrimental to innovation and contribution to knowledge.**

Reply: We have relied on previous studies making on this claim. For example, a great example is a study form van den Besselaar and Sandström (2015), where based on early career grant analysis they clearly bring out how “the grant decisions seem to have no predictive validity, as the successful applicants have no higher performance than the best performing non-successful applicants”. They do show how “the granted applicants have a much better academic career”, yet “the growing evidence that predictive validity of panel and peer review of grant applications is difficult to establish”. That said, grants help to rise in career ladder, yet do not predict future research excellence. With this we oppose the claim how there is lack of “past evidence to show that the standard approach to fund research (e.g., via individual peer-reviewers informing panels and the grant funders) is actually detrimental to innovation and contribution to knowledge.” Especially on the early career level.

**2.3. A second major issue is the fact that the data did not support the claims. Instead the data presented showed various descriptive facts about the grant funding allocations and a change in policy which had an impact on the type of applications received. By and large, the data provided a plausible picture: early career researchers get a grant when they are younger, with less papers although the actual numbers of papers vary with disciplines. Prior success in getting a grant is a strong predictor of future success, although this also varies across disciplines.**

Reply: The study revealed striking discipline based differences. For example, early career PIs may even outperform some advanced career PIs in terms of publications. That said, the seniority effect is rather weak. As we changed the focus of theoretical and problem setting anchor, the data now aligns well with the main findings.

**2.4. None of these data, however, directly provide evidence for the claims advanced. There is evidence that success rates fluctuates but this could also be due to the economic context rather than a deliberate attempt to increase competition. Not only that, success rate seems to have increased steadily since 2018. The evidence of a trend in increasing hyper-competitiveness is not compelling (especially since there are many possible causes for the fluctuations in success rates). Similarly there is no data to evidence the claim that allocations are random or that they are not supporting research excellence (however this may be defined). The reader is left asking themselves: "So what?" on many occasions.**

Reply: We have put the focus on early-career researchers, thus the hypercompetition is not a main claim in the paper. The main key takeaway is the RPRT effect in case of early-career researchers.

**2.5. A lot of the claims in the discussion seem unrelated to the evidence in the results section. For example "Our study confirmed the importance of luck in hypercompetitive research fund allocation" (lines 428-429). I do not see how the authors could infer this from the data they provided. One might as well say that the study "confirmed the importance of the excellence of the PI in hypercompetitive research fund allocation." Neither is implausible, neither can be substantiated by the data available. This is just an example. The same applies to all the claims in the discussion.**

Reply: We have detracted from anchoring ourselves around hypercompetition and luck throughout the paper. Problem statement, data and discussion are now much better aligned.

**2.6. The manuscript needs careful proofreading as it shows several instances of grammatical errors or sentences which would need rewriting to improve readability.**

Reply: The revised manuscript has gone through official English proofreading.

**2.7.The authors argue that the database they put together cannot be shared because of GDPR concerns. But I do not see why the authors could not share the anonymised data which underpin their graphs, especially if the data was collated from public sources.**

Reply: We will publish the anonymised data fully to the PLoS One.

Lastly, not signposted by the reviewers, but due to rather long waiting period (submitted on 24^th^ of may 2021, and reviews in January 2022), we could also add additional application year to the dataset. In the initial submission we covered 2013-2021 (9 years), but in current submission we cover 2013-2022 (10 years). That said, the updated database is even better than it was during initial submission.

**References**

Alberts, B., Kirschner, M.W., Tilghman, S., &Varmus, H. (2014). Rescuing US biomedical research from its systemic flaws. *PNAS*, 111(16): 5773–7.

Edwards, M. A., & Roy, S. (2017). Academic Research in the 21st Century: Maintaining Scientific Integrity in a Climate of Perverse Incentives and Hypercompetition. *Environmental Engineering Science*, 34(1): 51-61.

Engels, T.C.E., Ossenblok, T.L.B. & Spruyt, E.H.J. (2012). Changing publication patterns in the Social Sciences and Humanities, 2000–2009. *Scientometrics*, 93, 373–390.

Johann, D., & Mayer, S. J. (2018). The perception of scientific authorship across domains. *Minerva*, 57(2):175-196.

Mayer, S. J. 2016. Trends and developments in authorship patterns in five Social Science disciplines from 1991 to 2014. *Proceedings of the 21st international conference on science and technology indicators.*

Moore, S., Neylon, C., Paul Eve, M. et al. (2017). “Excellence R Us”: university research and the fetishisation of excellence. *Palgrave Commun*, 3.

Sahu, S. R., & Panda, K. C. (2014). Does the Multi-authorship Trend Influence the Quality of an Article? *Scientometrics*, 98(3), 2161-2168.

Smith, E., Williams-Jones, B., Master, Z., Larivière, V., Sugimoto, C. R., Paul-Hus, A., Shi, M., Diller, E., Caudle, K., & Resnik, D. B. (2020). Researchers' Perceptions of Ethical Authorship Distribution in Collaborative Research Teams. *Science and engineering ethics*, 26(4), 1995–2022.

Tol, R. S. J. (2011). Credit where credit's due: accounting for co-authorship in citation counts, *Scientometrics*, 89(1), 291-299.

van den Besselaar, P., & Sandström, U. (2015). Early career grants, performance, and careers: A study on predictive validity of grant decisions*. Journal of Informetrics*, 9(4): 826-838.

Watanabe, M. E. (2014). Unlikely Funding: Two years into a new application procedure, the NSF's DEB and IOS grants are scarce as hen's teeth. *BioScience*, 64(7): 563–568.
