# Supplementary material for: Getting funded in a highly fluctuating environment: Shifting from excellence to luck and timing
Source: PLoS One. 2022 Nov 7;17(11):e0277337. doi: 10.1371/journal.pone.0277337 (PMC9639839; doi:10.1371/journal.pone.0277337)
Supplement: Attachment — Submitted filename: Response to Reviewers.docx [file pone.0277337.s007.docx]

**RESPONSE TO REVIEWERS**

**EDITOR: The points to be addressed can be seen as minor in work required, but extremely important conceptually. Specifically, even though you have done consideration work in defining your conceptualisation of hyper-competition in the Estonian context, there are still problems with how your claims in the discussion are sufficiently supported by the results show.**

REPLY: We highly appreciate the feedback. In a revised version, we have given the above mentioned points remarkably more attention in the paper.

**EDITOR: In particular, I still find how you equate 'life experience' with 'academic experience' as underpinning your claims that how a swing towards success in grant allocation to older academics (arguably close to retirement) discriminations against young early-career researchers. Here, the use of the work 'discriminates' is problematic in that it assumes that older PhD graduates have an unfair advantage over younger ECRs.**

REPLY: We acknowledge the value-laden essence of the word “discrimination”. In the paper it is mentioned only in one place, under discussion:

“If grant allocation prefer older academics who have good record of articles, yet defended their doctoral degree around 50-60 years of age, this discriminates young early-career academics, who do not have such an advantage.” In a new version we have removed it.

To comment the claim “Here, the use of the work 'discriminates' is problematic in that it assumes that older PhD graduates have an unfair advantage over younger ECRs”, it is not so much the age, but the fact how older ECRs do have more publications, because they are often the academic staff who have been working in university without a degree, but doing research in a similar manner as other.

**EDITOR: Perhaps a more nuanced interpretation is that the funding agencies do not distinguish between older and younger ECRs based on age. This would focus your argument more appropriately on problematising the purpose of ECR funding, and the definition to an ECR, rather than assuming that there is a source of discrimination. Your findings support the former conclusion more robustly than the later.**

REPLY: We agree 100%. It is more based on established research track than merely on age. Yet, we see it to be not the problem of one characteristic, but a challenge that grounds on “intersectionality” phenomena. Age in our case correlates with long work experience in the academic system, thus having also good research track. That said, the age parameter interconnects with research productivity and so called insider advantage as compared to the young early career researchers who have been with the system only for let`s say 4-6 years ( as compared to 30 years). A person with 6 years experience and track in publishing is competing with a person with 30 years of experience.

We have now taken the focus away from the discrimination, but through the intersectionality phenomena we address the point how early career grants fail to fulfil their core mission – helping those who are at the very beginning at their career. In fact, the main difference between the early career and advanced career grant is the expected experience within the academic system and the publication track. People with 30 year experience are more eligible to advanced level competition with their research track, yet they apply for early career track, because there they can overshadow the young ERCs who haven’t got such an advantage.

**EDITOR: Adjusting the argument in the Abstract, introduction and conclusion would address this issue sufficiently.**

REPLY: In the revised version we have adjusted the argument not only in abstract, introduction and conclusion, but also in the discussion.

**EDITOR: I see this point as one of argument and for further debate, however this debate must extend from the paper. I would hate for any debate (of which the PLoS platform allows) to be distracted by the above issue, rather than contributing to a more robust problematisation of the function of the Estonian competitive grant and research system.**

REPLY: We strongly agree with the point. We see it as a question of our perhaps poor wording – as mentioned above, it is not a discrimination about the age, but a wider, intersectionality challenge. People with decades of experience and long list of publications are competing in the same pool with people who are literally new to the system. It undermines the mission of the early grant system, especially in case of a national research system that has very limited funding.

Best regards,

The authors
